# Molecular and Cytogenetic Study of East African Highland Banana

**DOI:** 10.3389/fpls.2018.01371

**Published:** 2018-10-04

**Authors:** Alžběta Němečková, Pavla Christelová, Jana Čížková, Moses Nyine, Ines Van den houwe, Radim Svačina, Brigitte Uwimana, Rony Swennen, Jaroslav Doležel, Eva Hřibová

**Affiliations:** ^1^Institute of Experimental Botany, Centre of the Region Haná for Biotechnological and Agricultural Research, Olomouc, Czechia; ^2^International Institute of Tropical Agriculture, Banana Breeding, Kampala, Uganda; ^3^Bioversity International, Banana Genetic Resources, Heverlee, Belgium; ^4^Division of Crop Biotechnics, Laboratory of Tropical Crop Improvement, Katholieke Universiteit Leuven, Leuven, Belgium; ^5^International Institute of Tropical Agriculture, Banana Breeding, Arusha, Tanzania

**Keywords:** East African highland bananas, fluorescence *in situ* hybridization (FISH), ITS phylogeny, *Musa*, rRNA genes, simple sequence repeats genotyping

## Abstract

East African highland bananas (EAHBs) are staple food crop in Uganda, Tanzania, Burundi, and other countries in the African Great Lakes region. Even though several morphologically different types exist, all EAHBs are triploid and display minimal genetic variation. To provide more insights into the genetic variation within EAHBs, genotyping using simple sequence repeat (SSR) markers, molecular analysis of ITS1-5.8S-ITS2 region of ribosomal DNA locus, and the analysis of chromosomal distribution of ribosomal DNA sequences were done. A total of 38 triploid EAHB accessions available in the *Musa* germplasm collection (International Transit Centre, Leuven, Belgium) were characterized. Six diploid accessions of *Musa acuminata* ssp. *zebrina*, ssp. *banksii*, and ssp. *malaccensis* representing putative parents of EAHBs were included in the study. Flow cytometric estimation of 2C nuclear DNA content revealed small differences (max ~6.5%) in genome size among the EAHB clones. While no differences in the number of 45S and 5S rDNA loci were found, genotyping using 19 SSR markers resulted in grouping the EAHB accessions into four clusters. The DNA sequence analysis of the internal transcribed spacer region indicated a relation of EAHB clones with *M. acuminata* and, surprisingly, also with *M. schizocarpa*. The results suggest that EAHB cultivars originated from a single hybrid clone with *M. acuminata* ssp. *zebrina* and ssp. *banksii* being its most probable parents. However, *M. schizocarpa* seems to have contributed to the formation of this group of banana.

## Introduction

Bananas and plantains are giant monocotyledonous plants of major importance in tropical and subtropical areas. Their domestication is not well-understood, but it is considered to have begun some 7000 years ago in Southeast Asia, which is considered the primary center of diversity from where bananas expanded to other parts of the world (D'Hont et al., 2012). Most of the edible banana cultivars are parthenocarpic triploid clones that originated from intra- and interspecific hybridization between subspecies of *Musa acuminata* with the A genome and *M. balbisiana* with the B genome. Among the nine subspecies of *M. acuminata* (*banksii, burmannica, burmannicoides, errans, malaccensis, microcarpa, siamea, truncata*, and *zebrina*), *banksii* is reported to have contributed to most of the domesticated bananas. New Guinea, where *banksii* originated, is considered to be the earliest and most active center of diversity for *Musa* (Perrier et al., 2009).

Based on genomic constitution, triploid edible banana clones are classified as AAA, AAB, and ABB and further assigned into subgroups based on morphological characteristics. It is believed that most of the modern edible triploids arose from their ancestors, brought by human migration to the secondary centers of diversification (e.g., Africa and Pacific Islands), where different clones evolved by accumulation of somatic mutations and selection by early farmers (Shepherd, 1957; Kitavi et al., 2016). Some edible clones are diploid or tetraploid and are believed to have originated in a similar way as the triploids. Minor groups of cultivated bananas are derived from interspecific hybridization of diploid *M. schizocarpa* (S genome) and *M. textilis* (T genome) in combination with *M. acuminata* and *M. balbisiana* (Carreel et al., 1994).

East African Highland bananas (EAHBs; genus *Musa*, family *Musaceae*, section Musa) are vegetatively propagated triploid clones also known as the Lujugira/Mutika subgroup with AAA genomic constitution. This subgroup is mostly grown in Burundi, Democratic Republic of Congo, Kenya, Rwanda, Tanzania, and Uganda, and consists of about 120 farmer-selected landrace cultivars. Despite their enormous socio-economic importance, little is known about their genetic variation and origin (Karamura, 1998; Ssebuliba et al., 2005, 2006), and almost no differences in their genetic diversity have been found using molecular tools (Kitavi et al., 2016; Christelová et al., 2017).

Recent studies suggest that the EAHBs evolved by clonal selection from one ancestor, and their phenotypic variability is a result of accumulation of somatic mutations (Crane and Lawrance, 1956; Shepherd, 1957; De Langhe, 1961; Ude et al., 2003). It is believed that the African Great Lakes region is a secondary center of *Musa* genetic diversity where phenotypic diversification of the EAHB took place (Tugume et al., 2002). Recent studies on the genetic diversity of EAHB indicated that diploid *M. acuminata* subspecies *zebrina* and *banksii* are putative parents of EAHBs (Li et al., 2013; Kitavi et al., 2016). A recent genotyping study by Christelová et al. (2017) using simple sequence repeat (SSR) markers on more than 600 representatives of wild diploid and cultivated triploid clones of bananas and plantains confirmed that *M. acuminata* subspecies *zebrina* and *banksii* as the closest relatives of EAHB clones.

The sequence of ITS1-5.8S-ITS2 region of ribosomal rRNA locus is highly polymorphic, and its analysis proved to be useful for resolving phylogenetic relationships in many plant species, including *Musa* spp. (e.g., Liu et al., 2010; Hřibová et al., 2011; Li et al., 2017). In *Musa*, ITS analysis facilitated identification of individual species as well as subspecies, and species-specific ITS types were found conserved in hybrid clones (Hřibová et al., 2011).

Unlike the analysis of genetic diversity using different types of molecular markers (Pillay et al., 2001; Christelová et al., 2017), chromosome studies on EAHB representatives are scarce. In this work, we performed a detailed cyto-molecular analysis of a set of 38 EAHB clones available from the International *Musa* Germplasm Transit Centre (https://www.bioversityinternational.org/banana-genebank/). A combination of flow cytometric and cytogenetic and molecular methods was used to (1) examine the variability in genome size; (2) determine genomic distribution of rRNA genes; (3) evaluate the relationships of EAHBs to other species within the *Musaceae* family; and (4) identify putative *Musa* species and subspecies that contributed to the evolution of EAHB clones. The multidisciplinary approach provided new information on genome organization and diversity of this important group of edible bananas.

## Materials and methods

### Plant material and genomic DNA extraction

The accessions of *Musa* analyzed in this work are listed in Table 1. *In vitro* rooted plants of 38 accessions representing the EAHB cultivars and six diploid subspecies of *M. acuminata* representing putative parents of EAHB were obtained from the International *Musa* Transit Centre (ITC, Katholieke Universiteit, Leuven, Belgium). The *in vitro* plants were transferred to soil, and all plants were maintained in a heated greenhouse.

**Table 1:** Nuclear DNA content, chromosome number, and the number of 45S and 5S rDNA loci and ITS diversity in East African highland banana (EAHB) and wild diploid species of *Musa acuminate*.

**EAHB Type**	**Genome**	**Accession name**	**ITC code**	**2C nuclear DNA content [pg], Mean ± SD**	**Monoploid genome size [Mb/1Cx]**	**ITS diversity θπ**	**Chromosome number (2n)**	45S rDNA*	5S rDNA^*^
Nfuka (cooking)	AAA	Guineo	0005	1.911 ± 0.010	623	40.211	33	3	6
Nfuka (cooking)	AAA	Kitawira	0137	1.934 ± 0.012	630	8.907	33	3	6
Nfuka (cooking)	AAA	Muhongoroka	0156	1.925 ± 0.017	628	36.176	33	3	6
Nfuka (cooking)	AAA	Ingarama	0160	1.858 ± 0.007	606	20.590	33	3	6
Nfuka (cooking)	AAA	Ingarama	0160	1.858 ± 0.007	606	20.590	33	3	6
Nfuka (cooking)	AAA	Bui Se-ed	0301	1.915 ± 0.006	624	30.691	33	3	6
Nfuka (beer)	AAA	Nshika	0145	1.935 ± 0.017	631	2.713	33	3	6
Nfuka (beer)	AAA	Naine de Nyangezi	0147	1.907 ± 0.009	622	3.141	33	3	6
Nfuka (beer)	AAA	Ikigeregere	0169	1.903 ± 0.023	620	18.980	33	3	6
Nfuka (beer)	AAA	Ikimaga	0171	1.929 ± 0.006	629	28.165	33	3	6
Nfuka	AAA	Imbogo	0168	1.903 ± 0.023	620	20.210	33	3	6
Nfuuka	AAA	Igisahira Gisanzwe (Inyamunyu)	0083	1.925 ± 0.007	628	3.802	33	—	—
Nfuuka	AAA	Inzirabahima	0150	1.924 ± 0.018	627	1.284	33	3	6
Nfuuka	AAA	Mbirabire	0154	1.919 ± 0.008	626	1.817	33	3	6
Nfuuka	AAA	Rugondo	0164	1.924 ± 0.012	627	5.190	33	3	6
Nfuuka	AAA	Bakurura	0170	1.898 ± 0.022	619	39.179	33	3	6
Nfuuka	AAA	N'Dundu	0732	1.945 ± 0.004	634	4.968	33	–	–
Nfuuka	AAA	Nante	1353	1.907 ± 0.007	622	13.855	33	3	6
Nakabululu (cooking)	AAA	Nyamahwa	1555	–	–	2.052	33	–	–
Nakabululu (cooking)	AAA	Nyitabunyonyi	1556	–	–	0.098	33	–	–
Nakabululu (beer)	AAA	Kikundi	1224	1.875 ± 0.008	611	–	33	3	6
Nakabululu	AAA	Nakitengwa	0085	1.888 ± 0.023	615	24.440	33	3	6
Nakabululu	AAA	Intama	0153	1.935 ± 0.014	631	4.593	33	3	6
Nakabululu	AAA	Intariho	0165	1.854 ± 0.004	604	4.323	33	3	6
Nakabululu	AAA	Nakitengwa	1180	1.906 ± 0.012	621	–	33	3	6
Nakabululu	AAA	Kazirakwe	1355	1.936 ± 0.009	631	36.039	33	3	6
Mbidde (beer)	AAA	Igitsiri (Intutu)	0081	1.815 ± 0.034	592	38.524	33	–	–
Mbidde (beer)	AAA	Ingumba y'lnyamunyo	0126	1.857 ± 0.036	605	47.122	33	–	–
Mbidde (beer)	AAA	Kagera	0141	1.931 ± 0.008	630	0.411	33	3	6
Mbidde (beer)	AAA	Gashulie	0149	1.895 ± 0.014	618	5.734	33	3	6
Mbidde (beer)	AAA	Ingumba y'lmbihire	0155	1.902 ± 0.008	620	19.808	33	3	6
Mbidde (beer)	AAA	Indemera y'lmbihire	0161	1.922 ± 0.010	627	3.024	33	3	6
Mbidde (beer)	AAA	Isha	0167	1.921 ± 0.010	626	38.506	33	3	6
Mbidde (beer)	AAA	Makara	0177	1.908 ± 0.013	622	23.593	33	3	6
Nakitembe	AAA	Nyamwihogora	0086	1.902 ± 0.018	620	38.668	33	–	–
Nakitembe	AAA	Igihuni	0158	1.911 ± 0.006	623	41.282	33	3	6
Nakitembe	AAA	Ingagara	0166	1.941 ± 0.008	633	2.453	33	3	6
Nakitembe	AAA	Mbwazirume	1356	–	–	1.379	33	–	–
Musakala	AAA	Inyoya	0163	1.914 ± 0.011	624	20.216	33	3	6
^**^	AA	M. acuminata 'Malaccensis'	0074	1.222 ± 0.011	598	14.846	22	2	6
^**^	AA	M. acuminata 'Zebrina'	1139	1.268 ± 0.006	620	26.604	22	2	4
^**^	AA	M. acuminata 'Zebrina'	1177	1.317 ± 0.001	644	23.772	22	2	4
^**^	AA	M. acuminata 'Monyeta'	1179	1.325 ± 0.006	648	21.801	22	2	4
^***^	AA	M. acuminata 'Kokopo 1'	1243	1.230 ± 0.006	601	8.000			
^***^	AA	M. acuminata 'Ndyali'	1552	1.251 ± 0.011	612	5.009	22	2	4

* *Number of FISH signals in a mitotic metaphase plate (2n)*.

** *Wild diploid*.

*** *Cultivated diploid*.

Genomic DNA was isolated from lyophilized leaves using NucleoSpin Plant II kit (Macherey-Nagel, Düren, Germany) following the manufacturer's recommendations.

### Flow cytometric analysis

Nuclear DNA content was estimated by flow cytometry according to Bartoš et al. (2005) and Čížková et al. (2015). *Glycine max* L. cv. Polanka (2C = 2.5 pg DNA; Doležel et al., 1994) served as the internal reference standard. The relative fluorescence intensity of propidium iodide-stained nuclei isolated from leaf tissues was analyzed using Partec PAS flow cytometer (Partec, Münster, Germany) equipped with a high-pressure mercury lamp as excitation light source. Five individuals were measured in each accession, each of them in three independent runs performed on different days. At least 5,000 nuclei were analyzed per sample and nuclear DNA content was calculated following the formula:

2C DNA content [pg] = 2.5 *x* G_1_ peak mean of *Musa* / G_1_ peak mean of *Glycine*

Mean DNA content of nuclei in the G1 phase of cell cycle (2C) was calculated for each accession, and monoploid genome size (1Cx) representing DNA content of a basic chromosome set x was determined considering 1 pg DNA equal to 0.978 × 10^9^ bp (Doležel et al., 2003).

### SSR genotyping

The SSR genotyping was performed using the pipeline established by Christelová et al. (2011). Briefly, 19 SSR loci (Crouch et al., 1998; Lagoda et al., 1998; Hippolyte et al., 2010) were amplified using a set of M13 tailed fluorescently labeled primers. Allele sizes were estimated using internal size standard (GeneScan^TM^-500 LIZ size standard; Applied Biosystems, Foster City, USA) on ABI 3730xl DNA analyzer (Applied Biosystems). The resulting data were analyzed using GeneMarker® v1.75 (Softgenetics, State College, USA), manually checked, and integrated into the existing database of *Musa* SSR profiles (core subset of accessions), which represents the subset of true-to-type *Musa* accessions verified by SSR genotyping (Christelová et al., 2017). For ease and clarity of interpretation, Callimusa accessions and triploid species with the B genome (AAB, ABB), which are part of the core subset (Christelová et al., 2017), were excluded from the final dendrogram. Genetic distances among individual accessions were calculated in PowerMarker v 3.25 (Liu and Muse, 2005) and hierarchical clustering analysis of resulting distance matrix was done using the UPGMA (Michener and Sokal, 1957). The output was visualized as a dendrogram using FigTree v1.4.0 (http://tree.bio.ed.ac.uk/software/figtree/). The SSR data are publically available at http://olomouc.ueb.cas.cz/projects/Musa/SSR and at Dryad Digital Repository: https://doi.org/10.5061/dryad.1759h94.

### Analysis of ITS1-5.8S-ITS2 region

The ITS sequence analysis was performed according to Hřibová et al. (2011). The ITS region was amplified from genomic DNA using polymerase chain reaction (PCR) with specific primers ITS-L and ITS-4 (Nwakanma et al., 2003). The PCR products were purified using ExoSAP-IT® (USB Corporation, Cleveland, USA) following the manufacturer's instructions, cloned into TOPO vector, and transformed into *Escherichia coli*-electrocompetent cells (Invitrogen Life Technologies, Carlsbad, USA). At least 48 and 96 cloned PCR products were sequenced in diploid and triploid accessions, respectively. The sequencing was carried out using the BigDye Terminator v3.1 Cycle Sequencing kit (Applied Biosystems) according to the manufacturer's instructions and run on ABI 3730xl DNA analyzer (Applied Biosystems). Nucleotide sequences were edited using Staden Package (Staden, 1996) and phylogenetic analysis was performed according to Hřibová et al. (2011). Sequence diversity was identified using DnaSAM program (Eckert et al., 2010) with 5,000 simulations. The sequences were aligned by MAFFT program v7.029 (–localpair –maxiterate 1000) (Katoh et al., 2005) and graphically displayed in SeaView v4.2.1 (Gouy et al., 2010). Datasets for this analysis comprised ITS sequences of the *Musa* accessions previously described in Hřibová et al. (2011) and Čížková et al. (2015). Phylograms were constructed based on Juke–Cantor distance matrix of the concatenated region containing ITS1 and ITS2 spacer sequences including putative pseudogenic sequences by BioNJ (Gascuel, 1997) and PhyML (Guindon and Gascuel, 2003) and by SplitsTree4 v4.1.11 (Huson and Bryant, 2006). The tree was rooted on *Ensete ventricosum* (ITC 1387). Nonparametric bootstrapping with 1000 pseudoreplicates was performed to assess the nodal support. Phylogenetic trees were drawn and edited using FigTree v1.4.0 (http://tree.bio.ed.ac.uk/software/figtree/). The ITS sequence alignments are publically available at http://olomouc.ueb.cas.cz/projects/Musa/ITS and at Dryad Digital Repository: https://doi.org/10.5061/dryad.1759h94.

### Chromosome preparation and fluorescence *in situ* hybridization (FISH)

Mitotic metaphase chromosome spreads were prepared according to Doleželová et al. (1998). Probes for 45S rDNA and 5S rDNA were prepared by labeling *Radka*1 (45S rDNA) and *Radka*2 (5S rDNA) DNA clones (Valárik et al., 2002) with digoxigenin-11-dUTP or biotin-16-dUTP (Roche Applied Science, Penzberg, Germany) using PCR with M13 forward and reverse primers (Invitrogen). Probes for tandem repeats CL18 and CL33 were amplified using specific primers (Hřibová et al., 2010) and labeled as the rDNA probes using PCR. Hybridization mixture consisting of 50% formamide, 10% dextran sulfate in 1×SSC, and 1 μg/ml of each labeled probe was added onto slides and denatured at 80°C for 3 min. The hybridization was carried out at 37°C overnight. The sites of probe hybridization were detected using anti-digoxigenin-FITC (Roche Applied Science) and streptavidin-Cy3 (Vector Laboratories, Burlingame, USA), and the chromosomes were counterstained with diamidino-2-phenylindole. The slides were examined with Axio Imager Z.2 Zeiss microscope (Zeiss, Oberkochen, Germany) equipped with Cool Cube 1 camera (Metasystems, Altlussheim, Germany) and appropriate optical filters. The capture of fluorescence signals and layers merging were performed with ISIS software (Metasystems); the final image adjustment was done in Adobe Photoshop 12.0.

## Results

### SSR genotyping

**Figure 1:**
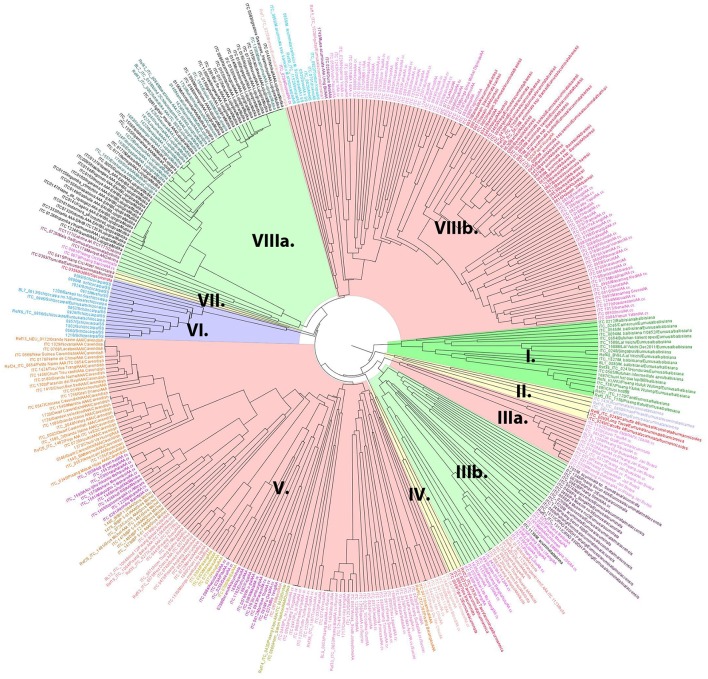
The UPGMA dendrogram constructed with SSR data of EAHB accessions obtained in this study, and the AA diploid and AAA triploid entries selected from the study of Christelová et al. (2011). *M. balbisiana* served as an outgroup species and was represented by a subset of *M. balbisiana* accessions (cluster I). Main clades and subclades are discriminated by colors. The EAHBs accessions analyzed in this study are included in cluster VIIIa (highlighted in green) and discriminated from the core subset accession by their names printed in black.

A dendrogram reflecting genetic diversity among the accessions was constructed after cluster analysis based on scores of 19 SSR markers (Christelová et al., 2011). The final dendrogram (Figure 1), which contains all EAHB representatives analyzed in this work, six diploid *M. acuminata* subspecies representing putative parents of EAHB, and the core subset of *Musa* accessions (Christelová et al., 2017) were adjusted as described in section Materials and Methods. The resulting tree showed clear groups of clustered accessions with the cluster of B-genome representatives (Figure 1, green cluster I. containing *M. balbisiana* samples) as outgroup. The clustering pattern was resolved after inspecting the dissimilarity index values (Nei, 1973). Values above 0.4 together with morphology-based classification track of the accessions extracted from the MGIS database (*Musa* Germplasm Information System; https://www.crop-diversity.org/mgis/) set the basis for fundamental division of the individuals into clusters and cluster description.

Wild A-genome representatives were interspersed among the related cultivated diploid and triploid accessions. Subspecies of *M. acuminata* ssp. *burmannicoides* and ssp. *siamea* were clustered within two small subclusters: subcluster II superimposed to the rest of the A-genome representatives (Figure 1, yellow cluster II) and subcluster IV. Cluster III comprised two subclusters (IIIa and IIIb). Subcluster IIIa (Figure 1, highlighted in light red) comprised diploid AA cultivars of the Pisang Jari Buaya subgroup, while subgroup IIIb (Figure 1, highlighted in light green) comprised *M. acuminata* ssp. *malaccensis* accessions grouped closely with triploid AAA subgroup Ibota representatives and diploid AA cultivars denoted as ISEA I group (Christelová et al., 2017). Except for Ibota and Lujugira/Mutika subgroups representing all EAHB accessions analyzed in this work, all other triploid AAA representatives formed cluster V (Figure 1, highlighted in light red). Apart from AAA cultivars Cavendish, Gros Michel, Ambon, Red, Rio, and Orotava, cluster V also contains diploid AA cultivars from groups denoted as AA cv. African, AA cv. ISEA 2, AA cv. IndonTriNG, AA cv. IndonTriPh (cluster names as described in Christelová et al., 2017). Accessions representing *M. schizocarpa* (S genome representatives) formed a separate cluster VI (Figure 1, highlighted in violet), followed by a small subcluster VII comprising *M. acuminata* ssp. *microcarpa* and *M. acuminata* ssp. *truncata*. Cluster VIII could be divided into two subclusters, where VIIIa (Figure 1, highlighted in green) contained the AAA EAHBs from subgroup Lujugira/Mutika, together with wild *M. acuminata* ssp. *zebrina* representatives and AA cv. Pisang Sapon accession, while the VIIIb subcluster (highlighted in light red) comprised subspecies *M. acuminata* ssp. *banksii* and their related AA cultivars (AA cv. *banksii sensu lato* and AA cv. *banksii* derivatives). Accessions representing hybrids between *M. acuminata* and *M. schizocarpa* species were also grouped within cluster VIIIb.

### Variability in genome size and cytogenetic analysis

**Figure 2:**
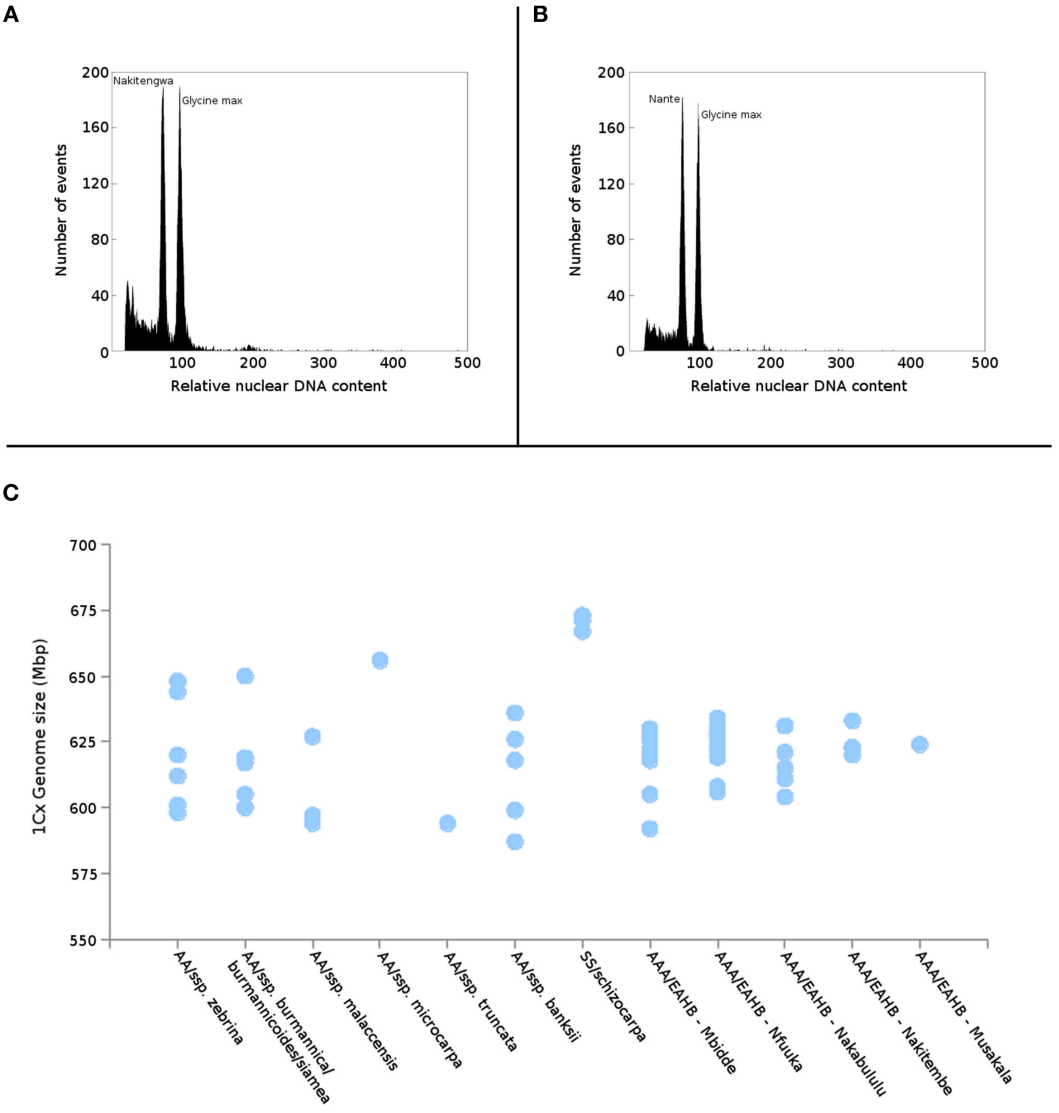
Estimation of genome size of EAHB. Histograms of relative nuclear DNA content obtained after flow cytometric analysis of propidium iodide-stained nuclei isolated from **(A)** “Nakitengwa” (2C = 1.888 pg) and **(B)** “Nante” (2C = 1.907 pg); nuclei isolated from soybean (*Glycine max*, 2C = 2.5 pg) were included as an internal reference standard. **(C)** Relationship between nuclear monoploid genome size (1Cx) in *M. acuminata* subspecies, *M. schizocarpa* estimated by Čížková et al. (2013) and five groups of EAHBs clones analyzed in the present study.

Nuclear genome size was estimated in all 38 EAHB accessions as well as in six subspecies of diploid *M. acuminata* selected to represent putative parents of EAHB clones (Table 1). Flow cytometric analyses resulted in histograms of relative nuclear DNA content (Figures 2A,B) comprising two dominant peaks representing G_1_ nuclei of *Musa* and *Glycine*, the latter serving as internal reference standard. Nuclear DNA content (2C value) was determined based on the ratio of G_1_ peak positions and ranged from 1.222 to 1.325 pg in diploid species (with the smallest DNA amount in *M. acuminata* ssp. *malaccensis* ITC 0074, and the largest in *M. acuminata* ssp. *zebrina* “Monyet” ITC 1179), and from 1.815 to 1.945 pg in triploid EAHB clones (Table 1). Thus, there was a difference of 0.13 pg between EAHB clones with the lowest and the highest 2C DNA amount. This corresponds to 42 Mbp/1Cx, where Cx stands for the monoploid genome size. Figure 2C shows 1Cx genome sizes in Mbp for all accessions analyzed.

**Figure 3:**
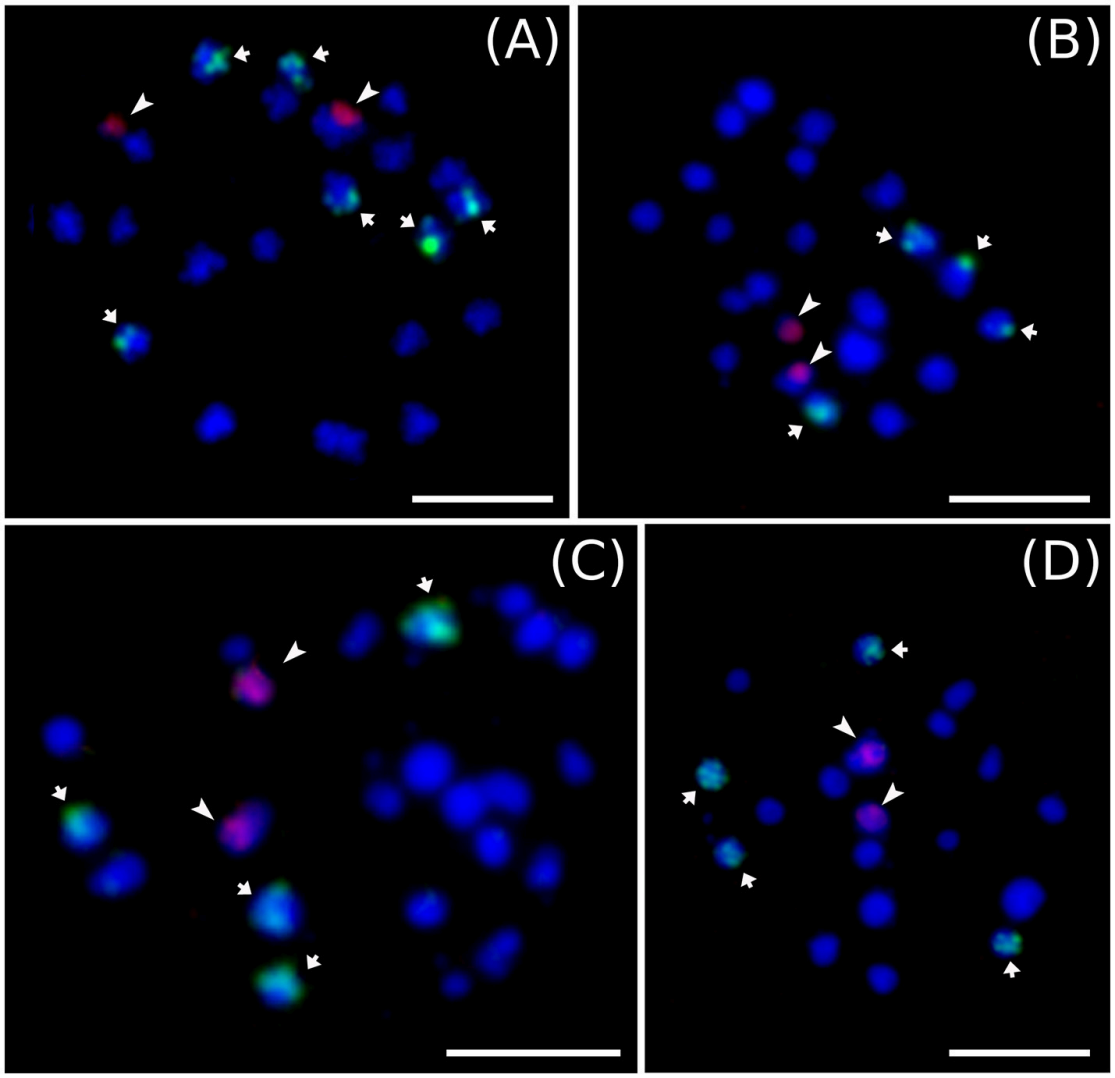
Examples of genomic distribution of 45S rRNA (green) and 5S rRNA (red) genes on mitotic metaphase chromosomes of diploid representatives of *M. acuminata* as revealed by FISH. **(A)** “Malaccensis” ITC 0074, **(B)** “Ndyali” ITC 1552, **(C)** “Monyeta” ITC 1179, and **(D)** “Zebrina” ITC 1177. Bar = 5 μm.

**Figure 4:**
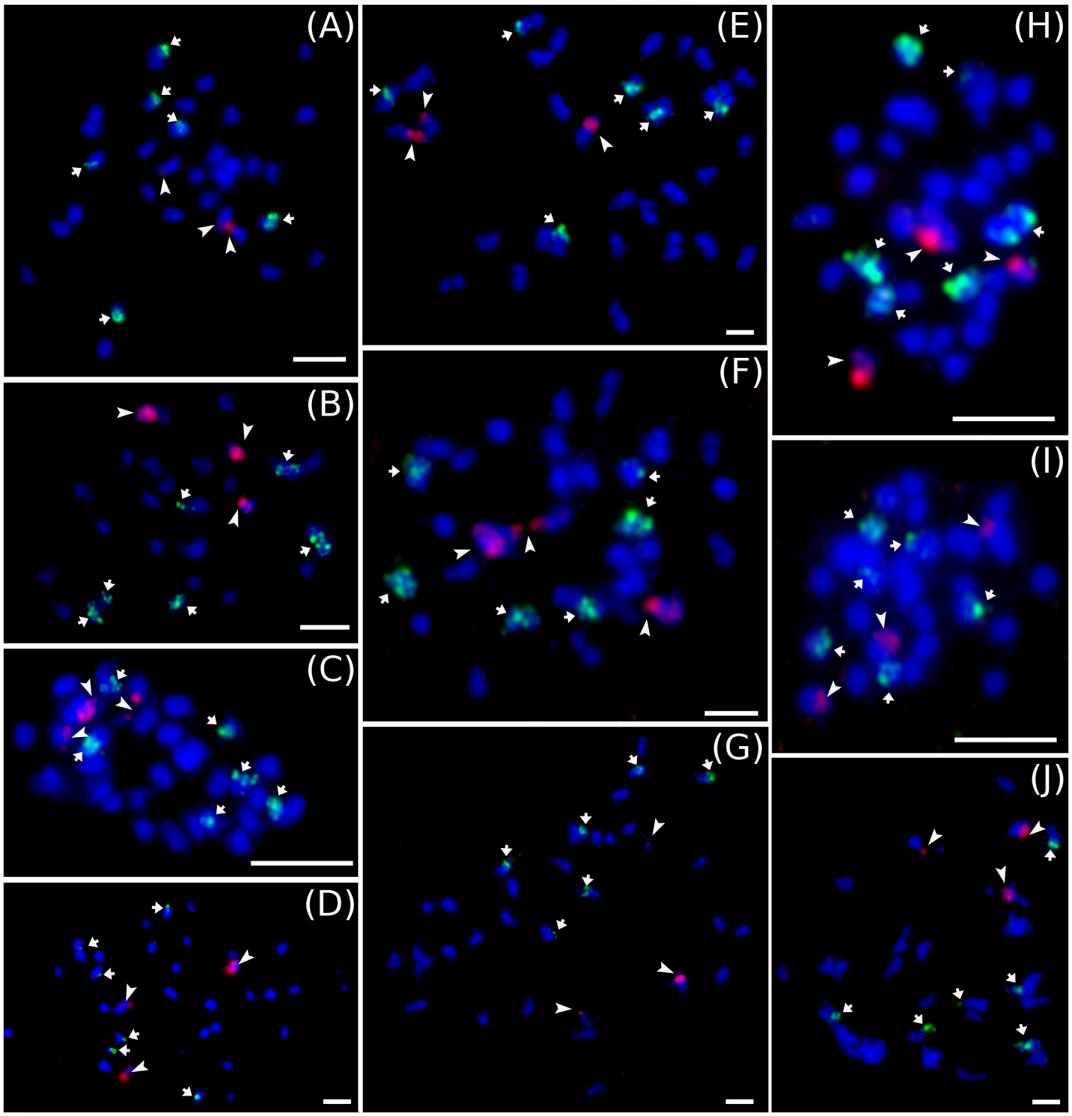
Examples of genomic distribution of 45S (red) and 5S rRNA (green) genes localized on mitotic metaphase chromosomes of EAHB as revealed by FISH. **(A)** “Nshika” ITC 0145, **(B)** “Bakurura” ITC 0170, **(C)** “Ingagara” ITC 0166, **(D)** “Naine de Nyangezi” ITC 0147, **(E)** “Kazirakwe” ITC 1355, **(F)** “Isha” ITC 0167, **(G)** “Guineo” ITC 0005, **(H)** “Muhongoroka” ITC 0156, **(I)** “Ikimaga” ITC 0171, **(J)** “Bui Se-ed” ITC 0301. Bar = 5 μm.

Cytogenetic localization of 5S and 45S rDNA on mitotic metaphase chromosomes of diploid *M. acuminata* species (Figure 3) revealed a constant number of 45S rDNA loci, which localized to nuclear organizing region (NOR) on one chromosome pair. On the contrary, variability was observed in the number 5S rDNA loci in diploid *Musa* accessions. *M. acuminata* ssp. *zebrina* (ITC 1139, ITC 1177, and ITC 1179) as well as diploid *M. acuminata* cultivar “Ndyali” (Mchare, ITC 1552) contained two pairs of 5S rDNA loci, while *M. acuminata* ssp. *malaccensis* (ITC 0074) contained three pairs of 5S rDNA loci (Figure 3). In contrast to the variability in the number of 5S rDNA loci among wild diploid *Musa* species, triploid EAHBs showed uniform pattern of rDNA loci organization. The 45S rDNA localized to NORs on three chromosomes and 5S rDNA localized on six chromosomes in all analyzed EAHB accessions (Figure 4).

### Analysis of ITS1-5.8S-ITS2 sequence region

The length of ITS1 and ITS2 spacers in all analyzed *Musa* accessions varied from 213 to 223 bp and from 205 to 219 bp, respectively, and the total length of ITS1-5.8S-ITS2 sequence region ranged from 566 to 593 bp in accessions. The lowest nucleotide diversity of the ITS1-5.8S-ITS2 sequence region was observed in EAHB clones “Nyitabunyonyi” (ITC 1556) and “Kagera” (ITC 0141) (Table 1). The highest sequence diversity was observed in EAHB accessions “Ingumba y'Imbihire” (ITC 0155), “Kazirakwe” (ITC 1355), “Muhongoroka” (ITC 0156), “Isha” (ITC 0167), “Igitsiri” (ITC 0081), “Nyamwihogora” (ITC 0086), “Bakurura” (ITC 0170), and “Igihuni” (ITC 0158). Relatively high sequence diversity of ITS region was observed in three accessions of *M. acuminata* ssp. z*ebrina* (ITC 1139, ITC1177, and ITC 1179) (Table 1, Supplementary Table 1). The GC content of ITS1 varied from 56.94 to 64.13% and was slightly lower than GC content of ITS2 (62.26 to 70.89%). The 5.8S rDNA sequence region had a conserved length of 154 or 155 bp and its GC content varied between 50.32 and 58.06%, and was significantly lower than the GC content in ITS1 and ITS2 based on Student's *t*-test (*P* < 0.001).

Prior to using the concatenated region of ITS1-ITS2 for phylogenetic analysis, secondary structures of ITS2 and 5.8S rDNA sequence regions were reconstructed for all accessions with the aim to identify putative pseudogenic sequences. The ITS2 sequences formed specific four-helices structure with typical pyrimidine–pyrimidine bulge in helix II and the most conserved primary sequence included TGGT motif in the helix III (Hřibová et al., 2011). The secondary structure of 5.8S rDNA sequence was reconstructed following specific settings for base pairing as described by Hřibová et al. (2011) and the presence of three conserved motifs in the 5.8S rRNA gene was identified (Harpke and Peterson, 2008). The information on nucleotide variation in conserved motifs of 5.8S rDNA; GC content; presence of conserved motifs in 5.8S rDNA sequence, and the ability of ITS2 and 5.8S rDNA sequences to fold conserved secondary structures was used to identify putative pseudogenes (Supplementary Table 1). The information on pseudogenic ITS sequences was used during further phylogenetic analysis.

**Figure 5:**
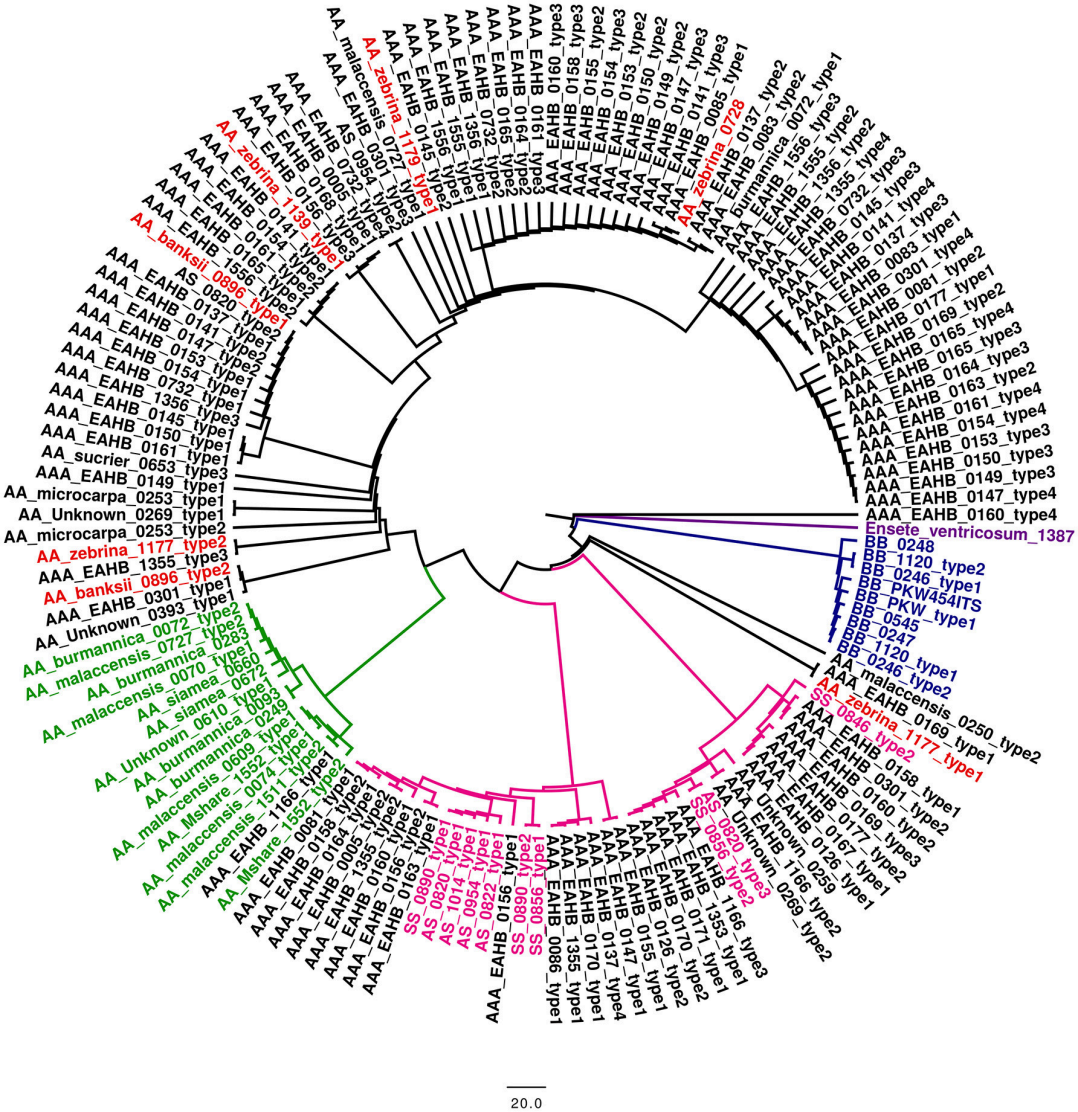
Phylogenetic analysis based on the ITS1-ITS2 sequence region. BioNJ tree constructed from a Jukes–Cantor distance matrix of the concatenated region contained ITS1 and ITS2 spacer sequence. The tree was rooted on *Ensete ventricosum* (ITC 1387). The main clades and subclades are distinguished by colors. The BB genotypes in blue; SS genotypes in pink; *burmannica/burmanicoides/siamea* and *malaccensis* subspecies of *M. acuminata* (AA genome) in green; *zebrina* and *banksii* subspecies of *M. acuminata* in red. ITS sequences of accessions analyzed in this study are shown in black.

Phylogenetic analysis of a dataset, which did not contain putative pseudogenic ITS sequences, was done by BioNj, PhyML, and SplitsTree (split decomposition). The analysis showed that ITS sequence types obtained from EAHB clones clustered together with the A genome-specific and S genome-specific ITS sequences (Figure 5). The ITS types obtained by clone-based sequencing in EAHB accessions did not cluster with *M. acuminata* subspecies *burmannica, burmannicoides* and *siamea*. Also, the cluster specific for *M. acuminata* ssp. *malaccensis* did not comprise ITS types from triploid EAHB clones. Phylogenetic analysis done on a dataset containing putative pseudogenic ITS sequences showed that most of them clustered together with ITS specific for *M. schizocarpa* genome (Supplementary Figure 1).

## Discussion

### Nuclear DNA content and distribution of rDNA

As shown by Poggio et al. (2014) and Duchoslav et al. (2013), nuclear genome size can be used to characterize individual accessions in mixed populations, especially in polyploid plants (Duchoslav et al., 2013; Poggio et al., 2014). In our study, we provide the first analysis of genome size in such a group of edible banana cultivars. The difference between EAHB clones with the lowest and the largest genome size was ~42 Mbp/1Cx (i.e., ~5% of EAHB monoploid genome; Figure 2). Considering the relatively small genome of *M. acuminata* (~ 600 Mbp/1C, Doležel et al., 1994), the difference corresponds to less than one chromosome. Importantly, the differences in genome size among the EAHB accessions were not significant enough to reveal any grouping. The occurrence of EAHB accessions with lower DNA content indicated a loss of DNA during the clonal selection from a common progenitor.

Although the evolution of EAHB clones remains obscure, based on previous studies (Karamura, 1998; Pillay et al., 2001; Li et al., 2013; Kitavi et al., 2016; Christelová et al., 2017) and the results obtained in the present study, it seems probable that the hybridization event that led to the formation of EAHB clones was followed by a loss of DNA sequences. If EAHB originated from a single ancestor, which came to Africa ~6000 years ago (Lejju et al., 2005, 2006), the variation in genome size among individual EAHB clones observed here would reflect genome changes accompanying secondary diversification.

In contrast to the variation in genome size, we did not observe differences in number of 45S and 5S rRNA chromosome loci (Figure 4). The 45S rDNA are localized to secondary constriction on three chromosomes and 5S rRNA gene clusters localized to six chromosomes in mitotic metaphase plates of all examined EAHB clones. In all clones, the large 45S rDNA subunit was on different mitotic chromosomes than the small 5S rDNA subunit. Due to the small size of condensed mitotic chromosomes (1–2 μm), it was not possible to ascertain if individual EAHB clones differed in chromosomal position of 5S rDNA loci. Thus, our results do not permit drawing any conclusions regarding the presence of structural chromosomal rearrangements.

The results of Boonruangrod et al. (2009), Perrier et al. (2009) and Hippolyte et al. (2012), pointed to *M. acuminata* ssp. *zebrina* and ssp. *banksii* as putative parents of EAHB clones. However, the number of 5S rDNA loci in EAHB clones, as determined in this work, does not correspond to the number of 5S rDNA loci expected in a triploid hybrid from a cross between *ssp. banksii* and ssp. *zebrina*, which have three and two 5S rDNA loci per haploid chromosome set, respectively (Bartoš et al., 2005; Čížková et al., 2013) (Supplementary Figure 2). Depending on the donor of unreduced gamete, the hybrid should have either seven or eight 5S rDNA loci. However, we have observed only six loci in all EAHB clones. While the same number of 5S rDNA loci in all EAHB clones indicates their origin from a single clone as proposed by Perrier et al. (2011) and Li et al. (2013), the discrepancy between the expected and observed number of 5S rDNA loci in EAHB can be explained either by a loss of 5S rDNA loci in a hybrid, different parent(s) than those indicated in previous studies, or a more complicated origin of EAHB involving more than two parental species. The latter hypothesis seems to be corroborated by our results on SSR genotyping and the presence of ITS sequences from *M. schizocarpa* in EAHB clones.

### SSR and ITS analysis

As mentioned earlier, based on morphological characterization, five main groups of EAHB can be recognized (Karamura, 1998). Unfortunately, these morphological groups do not correspond to the reported genetic diversity within EAHBs (Kitavi et al., 2016). The genetic variability of EAHB clones as analyzed in our study by SSR markers was very low (Nei's dissimilarity index < 0.16), which is in line with the previous results obtained on a set of Ugandan and Kenyan EAHB accessions (Kitavi et al., 2016) and on a small set of EAHB bananas analyzed by Christelová et al. (2017). The studies using SSR markers and other genotyping approaches, including genotyping by sequencing (GBS) and diversity array technology (DArT), failed to identify DNA markers discriminating the five morphological groups (Perrier et al., 2011; Hippolyte et al., 2012; Sardos et al., 2016). It this thus possible that epigenetic modifications played a role in the evolution and diversification of the triploid EAHB. Epigenetic changes, such as DNA methylation, might have affected the morphology of EAHB clones as shown in other plants, e.g., oil palm (Ong-Abdullah et al., 2015) and mangroves (Lira-Medeiros et al., 2010).

This work not only evaluated genetic diversity within EAHBs, but also expanded the database of *Musa* SSR profiles gathered during our long-term project aiming at genotyping all accessions in the International *Musa* Germplasm Transit Centre (Christelová et al., 2017). Addition of new accessions increases the resolution of genotyping, thereby, increasing a probability of identifying the closest relative or an exact match for unknown accession (Christelová et al., 2011, 2017; Čížková et al., 2015). The inclusion of the EAHB SSR profiles into the existing dataset of diploid *Musa* accessions suggested *M. acuminata* ssp. *zebrina* and ssp. *banksii* as parents of EAHB clones. At the same time, the SSR genotyping suggested that triploid EAHB clones did not evolve from East African diploids (Mchare cultivars with AA genome), which are common in Tanzania. These findings are in agreement with the recent studies of Hippolyte et al. (2012) and Sardos et al. (2016).

To ascertain the phylogenetic position of EAHB, we also analyzed nucleotide sequences of the ITS1-5.8S-ITS2 region (Alvarez and Wendel, 2003; Hřibová et al., 2011; Čížková et al., 2015). We also wanted to verify if the ITS region is suitable for unambiguous identification of the five morphological groups of EAHB (Karamura, 1998), which could not be resolved using SSR genotyping in our previous study (Christelová et al., 2017) and in the present work.

Previously, sequencing of ITS1-5.8S-ITS2 was employed to study phylogenetic relationships within the family (Li et al., 2010; Liu et al., 2010; Hřibová et al., 2011; Čížková et al., 2015) and characterize genomic constitution of intra- and interspecific hybrids (Hřibová et al., 2011). The latter studies showed that interspecific *Musa* hybrids contained conserved parental ITS sequences, indicating incomplete concerted evolution of rDNA loci. Independent evolution of parental rDNA in hybrids makes the analysis of ITS sequences suitable for determination of their genomic constitution.

Because of the polyploid status of EAHBs, we used clone-based sequencing, and at least 48 / 96 ITS clones from individual accessions were sequenced and used to identify ITS sequence types. Contrasting with the highly conserved pattern of genomic distribution of 45S rDNA as revealed by FISH, sequence analysis revealed relatively high variability of the ITS1-5.8S-ITS2 region in some of the EAHB representatives. Based on ITS region diversity, EAHB accessions could be classified into three groups: accessions with low ITS diversity (θπ ≤ 10), accessions with moderate ITS diversity (θπ > 10 & θπ ≤ 30), and accessions with relatively high level of ITS sequence diversity (θπ > 30, Table 1). This observation indicates a different evolutionary history of the locus. Unfortunately, the missing common ancestor of extant EAHB clones does not help to decide if the secondary diversification process in Africa led to homogenization of rDNA loci in some of the hybrids or vice versa.

The reconstruction of the ITS1-5.8S-ITS2 sequence region in triploid EAHB clones provided an opportunity to assess their relationships within Musaceae and identify putative parents. Phylogenetic reconstruction showed that EAHB clones do not contain *burmanicca/burmannicoides/siamea* ITS types, which is in agreement with the SSR genotyping. Moreover, ITS analysis did not confirm the presence of *malaccensis* ITS type, which is in agreement with our results obtained using SSR markers as well as the study by Perrier et al. (2009).

Almost all subspecies of *M. acuminata*, which were included in this study to enlarge the set of putative EAHB parents, clustered together with the respective subspecies of the core set both after SSR genotyping and ITS. The only exception being *M. acuminata* “Zebrina” ITC 1139, for which the results of SSR genotyping and ITS analysis did not agree with each other, suggesting a hybrid nature of the accession. While in the SSR cladogram, this accession clustered together with diploid *acuminata* representatives labeled as ISEA I and with *malaccensis* accessions, ITS analysis identified two ITS sequence types, one of them the *zebrina* type and one pseudogenic, thus pointing to a hybrid origin.

Our results indicate that the evolution of triploid EAHB clones was accompanied by a loss of rDNA loci and/or by homogenization of these loci. However, we cannot exclude one or more backcrosses to one of the parents during the evolution of EAHB (De Langhe et al., 2010), which would result in changes in the number and organization of rDNA loci. The analysis of the ITS region indicated the presence of *M. schizocarpa* ITS sequences in the genome of triploid EAHBs, pointing to a possible contribution of *M. schizocarpa* to EAHB formation. Participation of *M. schizocarpa* in the evolution of cultivated banana was suggested by Carreel et al. (2002) and Heslop-Harrison and Schwarzacher (2007). To conclude, the results of this study indicate that triploid *Musa* clones known as EAHBs, which are grown in the African Great Lakes region, probably arose from a single clone that originated from hybridization between *M. acuminata* ssp. *zebrina* and ssp. *banksii*, and that *M. schizocarpa* also contributed to the formation of this economically important group of banana.

## Author contributions

EH and JD designed the experiments. AN, PC, JČ, MN, and RSv conducted the study and processed the data. AN, PC, and EH wrote the manuscript. AN, PC, IV, RSw, BU, JD, and EH discussed the results and contributed to manuscript writing. All authors have read and approved the final manuscript.

### Conflict of interest statement

The authors declare that the research was conducted in the absence of any commercial or financial relationships that could be construed as a potential conflict of interest. The reviewer MB and handling editor declared their shared affiliation at the time of the review.

## Supplementary material

Supplementary Table 1 Sequence characterization of ITS1-5.8S-ITS2 sequence region.

Supplementary Figure 1 Phylogenetic analysis based on the ITS1-ITS2 sequence region. BioNJ tree constructed from a Jukes–Cantor distance matrix of the concatenated region contained ITS1 and ITS2 spacer sequence. The tree was rooted on *Ensete ventricosum* (ITC 1387). The main clades and subclades are distinguished by colors: BB genotypes in blue; SS genotypes in pink; *burmannica/burmanicoides/siamea* and *malaccensis* subspecies of *M. acuminata* (AA genome) in green; *zebrina* and *banksii* subspecies of *M. acuminata* in red. Putative pseudogenic ITS sequence regions are marked by asterisk.

Supplementary Figure 2 Scheme of genomic constitution of expected banana hybrid between *M. acuminata* ssp. *zebrina* and ssp. *banksii*. Red and violet marks in idiograms correspond to 45S rDNA and 5SrDNA loci, respectively.
