# Supplementary material for: Molecular and Cytogenetic Study of East African Highland Banana
Source: Front Plant Sci. 2018 Oct 4;9:1371. doi: 10.3389/fpls.2018.01371 (PMC6180188; doi:10.3389/fpls.2018.01371)
Supplement: Supplementary Table 1 — Sequence characterization of ITS1-5.8S-ITS2 sequence region. [file Table_1.DOCX]

**Supplementary Table 1. Sequence characteristics of ITS1-5.8S-ITS2 regions in *Musaceae***

| **Accession name** | **Label of ITS type^a^** | **ITS1^b^** | **5.8S^b^** | **ITS2^b^** | **Motif M1^c^** | **Motif M2^c^** | | **Motif M3^c^** | | **Secondary structure**  **of ITS2** | | **Secondary structure**  **of 5.8S** | | **Note** |
| --- | --- | --- | --- | --- | --- | --- | --- | --- | --- | --- | --- | --- | --- | --- |
| ***Musa acuminata* (AA)** |  |  |  |  |  |  |  | |  | |  | |  | |
| *Musa acuminata '*Ndyali' | 1552-type1 | 63.59 | 57.42 | 68.37 | conserved | conserved | conserved | | conserved | | conserved | |  | |
|  | 1552-type2 | 63.59 | 57.42 | 68.37 | conserved | conserved | conserved | | conserved | | conserved | |  | |
| *Musa acuminata '*Malaccensis' | 0074-type1 | 63.59 | 57.42 | 68.37 | conserved | conserved | conserved | | conserved | | conserved | |  | |
|  | ***0074-type2*** | ***59.74*** | ***53.55*** | ***64.49*** | ***conserved*** | ***conserved*** | ***conserved*** | | ***not formed*** | | ***conserved*** | | ***pseudogene*** | |
| *Musa acuminata* 'Monyet' | 1179-type1 | 62.04 | 57.42 | 69.77 | conserved | conserved | conserved | | conserved | | conserved | |  | |
|  | 1179-type2 | 60.19 | 53.90 | 64.79 | conserved | nt-14 'T' | conserved | | conserved | | conserved | |  | |
|  | ***1179-type3*** | ***59.26*** | ***54.19*** | ***62.26*** | ***conserved*** | ***nt-9 'T'*** | ***conserved*** | | ***not formed*** | | ***conserved*** | | ***pseudogene*** | |
| *Musa acuminata* 'Zebrina' | 1139-type1 | 60.65 | 55.48 | 65.89 | conserved | conserved | conserved | | conserved | | conserved | |  | |
|  | ***1139-type2*** | ***58.80*** | ***53.55*** | ***65.42*** | ***conserved*** | ***conserved*** | ***conserved*** | | ***not formed*** | | ***conserved*** | | ***pseudogene*** | |
| *Musa acuminata* 'Zebrina' | 1177-type1 | 58.33 | 54.84 | 62.26 | conserved | conserved | conserved | | conserved | | conserved | |  | |
|  | 1177-type2 | 60.19 | 53.90 | 69.48 | conserved | conserved | conserved | | conserved | | conserved | |  | |
|  | ***1177-type3*** | ***57.41*** | ***50.65*** | ***64.19*** | ***nt-12 'T'*** | ***conserved*** | ***conserved*** | | ***conserved*** | | ***conserved*** | | ***pseudogene*** | |
| *Musa acuminata* | 1243-type1 | 62.19 | 56.77 | 70.64 | conserved | conserved | conserved | | conserved | | conserved | |  | |
|  | 1243-type2 | 61.97 | 57.42 | 69.30 | conserved | conserved | conserved | | conserved | | conserved | |  | |
| **East African Highland Bananas (AAA)** |  |  |  |  |  |  |  | |  | |  | |  | |
| Guineo (Nfuka) | 0005-type1 | 61.11 | 52.90 | 66.36 | conserved | conserved | nt-6 'T' | | conserved | | conserved | |  | |
|  | 0005-type2 | 62.21 | 56.13 | 64.19 | nt-5 'A' | conserved | conserved | | conserved | | conserved | |  | |
|  | ***0005-type3*** | ***58.33*** | ***54.19*** | ***62.26*** | ***conserved*** | ***nt-9 'T'*** | ***conserved*** | | ***not formed*** | | ***conserved*** | | ***pseudogene*** | |
|  | ***0005-type4*** | ***56.94*** | ***52.26*** | ***64.02*** | ***nt-12 'A'*** | ***conserved*** | ***conserved*** | | ***not formed*** | | ***conserved*** | | ***pseudogene*** | |
|  | ***0005-type5*** | ***58.33*** | ***54.19*** | ***63.55*** | ***nt-11 'T'*** | ***conserved*** | ***conserved*** | | ***not formed*** | | ***conserved*** | | ***pseudogene*** | |
| Kitawira (Nfuka) | 0137-type1 | 63.08 | 57.42 | 70.87 | conserved | conserved | conserved | | conserved | | conserved | |  | |
|  | 0137-type2 | 62.04 | 57.42 | 70.05 | conserved | conserved | conserved | | conserved | | conserved | |  | |
|  | 0137-type3 | 61.75 | 57.42 | 69.91 | conserved | conserved | conserved | | conserved | | conserved | |  | |
|  | 0137-type4 | 63.13 | 57.42 | 70.32 | conserved | conserved | conserved | | conserved | | conserved | |  | |
|  | ***0137-type5*** | ***58.33*** | ***50.32*** | ***67.29*** | ***nt-11 'T'*** | ***nt-6 'A'*** | ***conserved*** | | ***conserved*** | | ***conserved*** | | ***pseudogene*** | |
| Muhongoroka (Nfuka) | 0156-type1 | 60.65 | 55.48 | 67.91 | conserved | nt-11 'G' | conserved | | conserved | | conserved | |  | |
|  | 0156-type2 | 63.89 | 56.13 | 68.84 | conserved | conserved | conserved | | conserved | | conserved | |  | |
|  | 0156-type3 | 62.96 | 57.42 | 69.16 | conserved | conserved | conserved | | conserved | | conserved | |  | |
|  | ***0156-type4*** | ***58.80*** | ***52.90*** | ***66.82*** | ***conserved*** | ***conserved*** | ***nt-4 'A'*** | | ***conserved*** | | ***not formed*** | | ***pseudogene*** | |
|  | ***0156-type5*** | ***58.33*** | ***54.84*** | ***63.72*** | ***conserved*** | ***nt-9 'T'*** | ***conserved*** | | ***not formed*** | | ***conserved*** | | ***pseudogene*** | |
|  | ***0156-type6*** | ***58.80*** | ***52.26*** | ***63.21*** | ***nt-16 'T'*** | ***nt-9 'T'*** | ***conserved*** | | ***not formed*** | | ***conserved*** | | ***pseudogene*** | |
|  | ***0156-type7*** | ***57.87*** | ***54.19*** | ***63.55*** | ***nt-11 'T'***  ***nt-16 'T'*** | ***nt-14 'T'*** | ***conserved*** | | ***not formed*** | | ***conserved*** | | ***pseudogene*** | |
|  | ***0156-type8*** | ***56.94*** | ***52.90*** | ***67.76*** | ***conserved*** | ***conserved*** | ***conserved*** | | ***not formed*** | | ***conserved*** | | ***pseudogene*** | |
| Ingarama (Nfuka) | 0160-type1 | 64.06 | 54.42 | 69.30 | conserved | conserved | conserved | | conserved | | conserved | |  | |
|  | 0160-type2 | 59.26 | 50.97 | 66.82 | nt-11 'T' | nt-6 'A' | conserved | | conserved | | conserved | |  | |
|  | 0160-type3 | 62.04 | 57.42 | 70.05 | conserved | conserved | conserved | | conserved | | conserved | |  | |
|  | 0160-type4 | 64.13 | 58.06 | 66.34 | conserved | conserved | conserved | | conserved | | conserved | |  | |
|  | ***0160-type5*** | ***57.87*** | ***55.48*** | ***69.63*** | ***nt-11 'T*** | ***nt-14 'T'*** | ***conserved*** | | ***conserved*** | | ***conserved*** | | ***pseudogene*** | |
|  | ***0160-type6*** | ***56.94*** | ***52.26*** | ***64.02*** | ***nt-12 'A'*** | ***conserved*** | ***conserved*** | | ***not formed*** | | ***conserved*** | | ***pseudogene*** | |
| Bui Se-ed (Nfuka) | 0301-type1 | 62.50 | 56.13 | 68.69 | conserved | conserved | conserved | | conserved | | conserved | |  | |
|  | 0301-type2 | 57.87 | 56.13 | 69.91 | conserved | conserved | conserved | | conserved | | conserved | |  | |
|  | 0301-type3 | 62.04 | 57.42 | 70.18 | conserved | conserved | conserved | | conserved | | conserved | |  | |
|  | 0301-type4 | 62.04 | 57.42 | 70.05 | conserved | conserved | conserved | | conserved | | conserved | |  | |
|  | ***0301-type5*** | ***57.87*** | ***54.19*** | ***63.55*** | ***nt-11 'T'***  ***nt-16 'T'*** | ***nt-14 'T'*** | ***conserved*** | | ***not formed*** | | ***conserved*** | | ***pseudogene*** | |
|  | ***0301-type6*** | ***56.94*** | ***52.26*** | ***63.68*** | ***nt-12 'A'*** | ***conserved*** | ***conserved*** | | ***not formed*** | | ***conserved*** | | ***pseudogene*** | |
| Nshika (Nfuka) | 0145-type1 | 62.91 | 57.42 | 69.91 | conserved | conserved | conserved | | conserved | | conserved | |  | |
|  | 0145-type2 | 62.04 | 57.42 | 68.84 | conserved | conserved | conserved | | conserved | | conserved | |  | |
|  | 0145-type3 | 61.57 | 57.42 | 70.05 | conserved | conserved | conserved | | conserved | | conserved | |  | |
| Naine de Nyangezi (Nfuka) | 0147-type1 | 62.96 | 57.42 | 69.91 | conserved | conserved | conserved | | conserved | | conserved | |  | |
|  | 0147-type2 | 62.61 | 57.42 | 70.64 | conserved | conserved | conserved | | conserved | | conserved | |  | |
|  | 0147-type3 | 62.04 | 57.42 | 70.05 | conserved | conserved | conserved | | conserved | | conserved | |  | |
|  | 0147-type4 | 61.57 | 57.42 | 69.91 | conserved | conserved | conserved | | conserved | | conserved | |  | |
| Ikigeregere (Nfuka) | 0169-type1 | 61.57 | 57.42 | 69.81 | conserved | conserved | conserved | | conserved | | conserved | |  | |
|  | 0169-type2 | 61.57 | 57.42 | 69.91 | conserved | conserved | conserved | | conserved | | conserved | |  | |
|  | 0169-type3 | 59.26 | 57.42 | 70.09 | conserved | conserved | conserved | | conserved | | conserved | |  | |
|  | ***0169-type2*** | ***57.87*** | ***54.84*** | ***70.37*** | ***nt-11 'T'***  ***nt-16 'T'*** | ***conserved*** | ***conserved*** | | ***conserved*** | | ***conserved*** | | ***pseudogene*** | |
|  | ***0169-type5*** | ***56.94*** | ***52.26*** | ***64.02*** | ***nt-12 'A'*** | ***conserved*** | ***conserved*** | | ***not formed*** | | ***conserved*** | | ***pseudogene*** | |

**Table 3. continued....**

| **Accession name** | **Label of ITS type^a^** | **ITS1^b^** | **5.8S^b^** | **ITS2^b^** | **Motif M1^c^** | **Motif M2^c^** | **Motif M3^c^** | **Secondary structure**  **of ITS2** | **Secondary structure**  **of 5.8S** | **Note** |
| --- | --- | --- | --- | --- | --- | --- | --- | --- | --- | --- |
| **East African Highland Bananas (AAA)** |  |  |  |  |  |  |  |  |  |  |
| Ikimaga (Nfuka) | 0171-type1 | 57.87 | 54.19 | 63.55 | nt-11 'T'  nt-16 'T' | nt-14 'T' | conserved | conserved | conserved |  |
|  | ***0171-type2*** | ***58.33*** | ***54.19*** | ***62.26*** | ***conserved*** | ***nt-9 'T'*** | ***conserved*** | ***not formed*** | ***conserved*** | ***pseudogene*** |
|  | ***0171-type3*** | ***56.02*** | ***54.84*** | ***65.42*** | ***conserved*** | ***nt-9 'T'*** | ***conserved*** | ***not formed*** | ***conserved*** | ***pseudogene*** |
|  | ***0171-type4*** | ***56.94*** | ***52.26*** | ***64.02*** | ***nt-12 'A'*** | ***conserved*** | ***conserved*** | ***not formed*** | ***conserved*** | ***pseudogene*** |
| Imbogo (Nfuka) | 0168-type1 | 62.04 | 58.06 | 69.16 | conserved | conserved | conserved | conserved | conserved |  |
|  | ***0168-type2*** | ***57.87*** | ***54.19*** | ***63.55*** | ***nt-11 'T'***  ***nt-16 'T'*** | ***nt-14 'T'*** | ***conserved*** | ***conserved*** | ***conserved*** | ***pseudogene*** |
|  | ***0168-type3*** | ***58.33*** | ***54.19*** | ***65.09*** | ***conserved*** | ***nt-9 'T'*** | ***conserved*** | ***not formed*** | ***conserved*** | ***pseudogene*** |
|  | ***0168-type4*** | ***59.26*** | ***52.26*** | ***63.21*** | ***conserved*** | ***conserved*** | ***conserved*** | ***not formed*** | ***conserved*** | ***pseudogene*** |
|  | ***0168-type5*** | ***55.09*** | ***52.26*** | ***65.89*** | ***conserved*** | ***nt-7 'T'*** | ***nt-4 'A'*** | ***not formed*** | ***not formed*** | ***pseudogene*** |
|  | ***0168-type6*** | ***56.94*** | ***52.26*** | ***64.02*** | ***nt-12 'A'*** | ***conserved*** | ***conserved*** | ***not formed*** | ***conserved*** | ***pseudogene*** |
| Igisahira Gisanzwe (Inyamunyu) | 0083-type1 | 61.57 | 57.42 | 69.91 | conserved | conserved | conserved | conserved | conserved |  |
|  | 0083-type2 | 62.04 | 57.42 | 69.91 | conserved | conserved | conserved | conserved | conserved |  |
|  | ***0083-type3*** | ***62.50*** | ***57.42*** | ***69.91*** | ***conserved*** | ***conserved*** | ***conserved*** | ***not formed*** | ***conserved*** | ***pseudogene*** |
| Inzirabahima (Nfuka) | 0150-type1 | 62.91 | 57.42 | 69.91 | conserved | conserved | conserved | conserved | conserved |  |
|  | 0150-type2 | 62.04 | 57.42 | 70.05 | conserved | conserved | conserved | conserved | conserved |  |
|  | 0150-type3 | 61.57 | 57.42 | 69.91 | conserved | conserved | conserved | conserved | conserved |  |
| Mbirabire (Nfuka) | 0154-type1 | 62.91 | 57.42 | 70.78 | conserved | conserved | conserved | conserved | conserved |  |
|  | 0154-type2 | 62.96 | 57.42 | 70.05 | conserved | conserved | conserved | conserved | conserved |  |
|  | 0154-type3 | 62.04 | 57.42 | 70.05 | conserved | conserved | conserved | conserved | conserved |  |
|  | 0154-type4 | 61.57 | 57.42 | 70.05 | conserved | conserved | conserved | conserved | conserved |  |
| Rugondo (Nfuka) | 0164-type1 | 62.04 | 56.13 | 68.84 | conserved | conserved | conserved | conserved | conserved |  |
|  | 0164-type2 | 62.04 | 57.42 | 70.32 | conserved | conserved | conserved | conserved | conserved |  |
|  | 0164-type3 | 61.57 | 57.42 | 69.91 | conserved | conserved | conserved | conserved | conserved |  |
| Bakurura (Nfuka) | 0170-type1 | 62.96 | 57.42 | 69.16 | conserved | conserved | conserved | conserved | conserved |  |
|  | 0170-type2 | 57.87 | 54.19 | 63.55 | nt-11 'T'  nt-16 'T' | nt-14 'T' | conserved | conserved | conserved |  |
|  | ***0170-type3*** | ***58.33*** | ***54.19*** | ***62.26*** | ***conserved*** | ***nt-9 'T'*** | ***conserved*** | ***not formed*** | ***conserved*** | ***pseudogene*** |
|  | ***0170-type4*** | ***59.26*** | ***52.26*** | ***63.21*** | ***nt-16 'T'*** | ***nt-9 'T'*** | ***conserved*** | ***not formed*** | ***conserved*** | ***pseudogene*** |
|  | ***0170-type5*** | ***56.94*** | ***52.26*** | ***64.02*** | ***nt-12 'A'*** | ***conserved*** | ***conserved*** | ***not formed*** | ***conserved*** | ***pseudogene*** |
| N'Dundu (Nfuka) | 0732-type1 | 62.91 | 57.42 | 70.78 | conserved | conserved | conserved | conserved | conserved |  |
|  | 0732-type2 | 62.04 | 57.42 | 70.18 | conserved | conserved | conserved | conserved | conserved |  |
|  | 0732-type3 | 61.57 | 57.42 | 70.05 | conserved | conserved | conserved | conserved | conserved |  |
|  | 0732-type4 | 62.50 | 57.42 | 69.59 | conserved | conserved | conserved | conserved | conserved |  |
| Nante (Nfuka) | 1353-type1 | 57.87 | 54.19 | 63.55 | nt-11 'T'  nt-16 'T' | nt-14 'T' | conserved | conserved | conserved |  |
|  | ***1353-type2*** | ***55.09*** | ***52.26*** | ***65.42*** | ***conserved*** | ***nt-7 'T'*** | ***nt-4 'A'*** | ***conserved*** | ***not formed*** | ***pseudogene*** |
|  | ***1353-type3*** | ***58.33*** | ***54.19*** | ***62.26*** | ***conserved*** | ***nt-9 'T'*** | ***conserved*** | ***not formed*** | ***conserved*** | ***pseudogene*** |
|  | ***1353-type4*** | ***56.94*** | ***52.26*** | ***64.02*** | ***nt-12 'A'*** | ***conserved*** | ***conserved*** | ***not formed*** | ***conserved*** | ***pseudogene*** |
| Nyamahwa (Nakabululu) | 1555-type1 | 62.04 | 57.42 | 70.05 | conserved | conserved | conserved | conserved | conserved |  |
|  | 1555-type2 | 61.57 | 57.42 | 70.05 | conserved | conserved | conserved | conserved | conserved |  |
|  | ***1555-type3*** | ***62.91*** | ***57.42*** | ***70.05*** | ***conserved*** | ***nt-9 'T'*** | ***conserved*** | ***not formed*** | ***conserved*** | ***pseudogene*** |
| Nyitabunyonyi (Nakabululu) | 1556-type1 | 62.04 | 57.42 | 70.05 | conserved | conserved | conserved | conserved | conserved |  |
|  | 1556-type2 | 62.50 | 57.42 | 70.05 | conserved | conserved | conserved | conserved | conserved |  |
|  | 1556-type3 | 61.57 | 57.42 | 70.05 | conserved | conserved | nt-4 'A' | conserved | conserved |  |
| Nakitengwa (Nakabululu) | 0085-type1 | 62.04 | 57.42 | 70.05 | conserved | conserved | conserved | conserved | conserved |  |
|  | ***0085-type2*** | ***59.26*** | ***52.26*** | ***63.21*** | ***nt-16 'T'*** | ***nt-9 'T'*** | ***conserved*** | ***not formed*** | ***conserved*** | ***pseudogene*** |
|  | ***0085-type3*** | ***58.33*** | ***54.19*** | ***62.26*** | ***conserved*** | ***nt-9 'T'*** | ***conserved*** | ***not formed*** | ***conserved*** | ***pseudogene*** |
|  | ***0085-type4*** | ***56.94*** | ***52.26*** | ***64.02*** | ***nt-12 'A'*** | ***conserved*** | ***conserved*** | ***not formed*** | ***conserved*** | ***pseudogene*** |
| Intama (Nakabululu) | 0153-type1 | 62.91 | 57.42 | 70.32 | conserved | conserved | conserved | conserved | conserved |  |
|  | 0153-type2 | 62.04 | 57.42 | 69.91 | conserved | conserved | conserved | conserved | conserved |  |
|  | 0153-type3 | 61.57 | 57.42 | 70.18 | conserved | conserved | conserved | conserved | conserved |  |
| Intariho (Nakabululu) | 0165-type1 | 62.96 | 57.42 | 69.91 | conserved | conserved | conserved | conserved | conserved |  |
|  | 0165-type2 | 62.04 | 57.42 | 70.05 | conserved | conserved | conserved | conserved | conserved |  |
|  | 0165-type3 | 61.57 | 57.42 | 69.91 | conserved | conserved | conserved | conserved | conserved |  |
|  | 0165-type4 | 61.75 | 57.42 | 70.05 | conserved | conserved | conserved | conserved | conserved |  |

**Table 3. continued....**

| **Accession name** | **Label of ITS type^a^** | **ITS1^b^** | **5.8S^b^** | **ITS2^b^** | **Motif M1^c^** | **Motif M2^c^** | **Motif M3^c^** | **Secondary structure**  **of ITS2** | **Secondary structure**  **of 5.8S** | **Note** |
| --- | --- | --- | --- | --- | --- | --- | --- | --- | --- | --- |
| **East African Highland Bananas (AAA)** |  |  |  |  |  |  |  |  |  |  |
| Kazirakwe (Nakabululu) | 1355-type1 | 63.89 | 56.77 | 68.69 | conserved | conserved | conserved | conserved | conserved |  |
|  | 1355-type2 | 62.21 | 56.13 | 64.19 | nt-5 'A' | conserved | conserved | conserved | conserved |  |
|  | 1355-type3 | 59.72 | 52.90 | 65.73 | nt-15 'A' | conserved | conserved | conserved | conserved |  |
|  | 1355-type4 | 61.57 | 57.42 | 69.91 | conserved | conserved | conserved | conserved | conserved |  |
|  | ***1355-type5*** | ***57.87*** | ***54.19*** | ***63.55*** | ***nt-11 'T'***  ***nt-16 'T'*** | ***nt-14 'T'*** | ***conserved*** | ***conserved*** | ***conserved*** | ***pseudogene*** |
|  | ***1355-type6*** | ***56.04*** | ***52.26*** | ***66.36*** | ***conserved*** | ***nt-7 'T'*** | ***nt-4 'A'*** | ***conserved*** | ***not formed*** | ***pseudogene*** |
|  | ***1355-type7*** | ***58.33*** | ***54.19*** | ***62.26*** | ***conserved*** | ***nt-9 'T'*** | ***conserved*** | ***not formed*** | ***conserved*** | ***pseudogene*** |
|  | ***1355-type8*** | ***59.26*** | ***52.26*** | ***63.21*** | ***nt-16 'T'*** | ***nt-9 'T'*** | ***conserved*** | ***not formed*** | ***conserved*** | ***pseudogene*** |
|  | ***1355-type9*** | ***56.94*** | ***52.26*** | ***64.02*** | ***nt-12 'A'*** | ***conserved*** | ***conserved*** | ***not formed*** | ***conserved*** | ***pseudogene*** |
| Igitsiri (Intutu) (Mbidde) | 0081-type1 | 62.50 | 56.77 | 65.58 | conserved | conserved | conserved | conserved | conserved |  |
|  | 0081-type2 | 61.57 | 57.42 | 69.91 | conserved | conserved | conserved | conserved | conserved |  |
|  | ***0081-type3*** | ***56.94*** | ***52.26*** | ***64.02*** | ***nt-12 'A'*** | ***conserved*** | ***conserved*** | ***not formed*** | ***conserved*** | ***pseudogene*** |
|  | ***0081-type4*** | ***58.33*** | ***54.19*** | ***62.26*** | ***conserved*** | ***nt-14 'T'*** | ***conserved*** | ***not formed*** | ***conserved*** | ***pseudogene*** |
|  | ***0081-type5*** | ***59.26*** | ***52.26*** | ***63.21*** | ***conserved*** | ***nt-9 'T'*** | ***conserved*** | ***not formed*** | ***conserved*** | ***pseudogene*** |
|  | ***0081-type6*** | ***57.87*** | ***54.19*** | ***63.55*** | ***nt-11 'T'***  ***nt-16 'T'*** | ***nt-14 'T'*** | ***conserved*** | ***not formed*** | ***conserved*** | ***pseudogene*** |
| Ingumba y'lnyamunyo (Mbidde) | 0126-type1 | 59.26 | 52.26 | 64.02 | nt-12 'A' | conserved | conserved | conserved | conserved |  |
|  | 0126-type2 | 57.87 | 54.19 | 63.55 | nt-11 'T'  nt-16 'T' | nt-14 'T' | conserved | conserved | conserved |  |
|  | ***0126-type3*** | ***58.33*** | ***54.19*** | ***62.26*** | ***conserved*** | ***nt-9 'T'*** | ***conserved*** | ***not formed*** | ***conserved*** | ***pseudogene*** |
|  | ***0126-type4*** | ***56.94*** | ***52.26*** | ***63.21*** | ***nt-16 'T'*** | ***nt-9 'T'*** | ***conserved*** | ***not formed*** | ***conserved*** | ***pseudogene*** |
| Kagera (Mbidde) | 0141-type1 | 63.43 | 57.42 | 69.91 | conserved | conserved | conserved | conserved | conserved |  |
|  | 0141-type2 | 62.91 | 57.42 | 70.87 | conserved | conserved | conserved | conserved | conserved |  |
|  | 0141-type3 | 62.04 | 57.42 | 70.05 | conserved | conserved | conserved | conserved | conserved |  |
|  | 0141-type4 | 61.75 | 57.42 | 69.91 | conserved | conserved | conserved | conserved | conserved |  |
| Gashulie (Mbidde) | 0149-type1 | 62.50 | 57.42 | 70.78 | conserved | conserved | conserved | conserved | conserved |  |
|  | 0149-type2 | 62.04 | 57.42 | 69.91 | conserved | conserved | conserved | conserved | conserved |  |
|  | 0149-type3 | 61.57 | 57.42 | 69.91 | conserved | conserved | conserved | conserved | conserved |  |
| Ingumba y'lmbihire (Mbidde) | 0155-type1 | 63.43 | 57.42 | 70.09 | conserved | conserved | conserved | conserved | conserved |  |
|  | 0155-type2 | 62.04 | 57.42 | 70.05 | conserved | conserved | conserved | conserved | conserved |  |
|  | ***0155-type3*** | ***58.33*** | ***54.19*** | ***62.26*** | ***conserved*** | ***nt-9 'T'*** | ***conserved*** | ***not formed*** | ***conserved*** | ***pseudogene*** |
| Indemera y'lmbihire (Mbidde) | 0161-type1 | 62.91 | 57.42 | 70.18 | conserved | conserved | conserved | conserved | conserved |  |
|  | 0161-type2 | 62.50 | 57.42 | 69.91 | conserved | conserved | conserved | conserved | conserved |  |
|  | 0161-type3 | 62.04 | 57.42 | 69.91 | conserved | conserved | conserved | conserved | conserved |  |
|  | 0161-type4 | 61.57 | 57.42 | 69.91 | conserved | conserved | conserved | conserved | conserved |  |
| Isha (Mbidde) | 0167-type1 | 59.26 | 52.26 | 63.21 | nt-16 'T' | nt-9 'T' | conserved | conserved | conserved |  |
|  | ***0167-type2*** | ***56.94*** | ***52.26*** | ***64.02*** | ***nt-12 'A'*** | ***conserved*** | ***conserved*** | ***not formed*** | ***conserved*** | ***pseudogene*** |
|  | ***0167-type3*** | ***58.33*** | ***54.19*** | ***62.26*** | ***conserved*** | ***nt-9 'T'*** | ***conserved*** | ***not formed*** | ***conserved*** | ***pseudogene*** |
| Makara (Mbidde) | 0177-type1 | 61.57 | 57.42 | 69.91 | conserved | conserved | conserved | conserved | conserved |  |
|  | 0177-type2 | 56.94 | 52.26 | 64.02 | nt-12 'A' | conserved | conserved | conserved | conserved |  |
|  | ***0177-type3*** | ***59.72*** | ***52.26*** | ***63.21*** | ***nt-16 'T'*** | ***nt-9 'T'*** | ***conserved*** | ***not formed*** | ***conserved*** | ***pseudogene*** |
|  | ***0177-type4*** | ***58.33*** | ***54.19*** | ***62.26*** | ***conserved*** | ***nt-9 'T'*** | ***conserved*** | ***not formed*** | ***conserved*** | ***pseudogene*** |
| Nyamwihogora (Nakitembe) | 0086-type1 | 62.96 | 57.42 | 69.77 | conserved | conserved | conserved | conserved | conserved |  |
|  | ***0086-type2*** | ***57.41*** | ***52.26*** | ***64.02*** | ***nt-12 'A'*** | ***conserved*** | ***conserved*** | ***conserved*** | ***conserved*** | ***pseudogene*** |
|  | ***0086-type3*** | ***58.33*** | ***54.19*** | ***62.26*** | ***conserved*** | ***nt-9 'T'*** | ***conserved*** | ***not formed*** | ***conserved*** | ***pseudogene*** |
|  | ***0086-type4*** | ***59.26*** | ***52.26*** | ***63.21*** | ***nt-16 'T'*** | ***nt-9 'T'*** | ***conserved*** | ***not formed*** | ***conserved*** | ***pseudogene*** |
|  | ***0086-type5*** | ***58.33*** | ***54.19*** | ***63.55*** | ***nt-11 'T'***  ***nt-16 'T'*** | ***nt-14 'T'*** | ***conserved*** | ***not formed*** | ***conserved*** | ***pseudogene*** |
|  | ***0086-type6*** | ***56.94*** | ***5032*** | ***63.21*** | ***nt-12 'A'*** | ***nt-9 'T'*** | ***conserved*** | ***not formed*** | ***conserved*** | ***pseudogene*** |
| Igihuni (Nakitembe) | 0158-type1 | 61.11 | 56.77 | 70.09 | conserved | conserved | conserved | conserved | conserved |  |
|  | 0158-type2 | 62.50 | 56.49 | 65.58 | conserved | conserved | conserved | conserved | conserved |  |
|  | 0158-type3 | 62.04 | 57.42 | 69.91 | conserved | conserved | conserved | conserved | conserved |  |
|  | ***0158-type4*** | ***57.87*** | ***54.19*** | ***64.49*** | ***nt-11 'T'***  ***nt-16 'T'*** | ***nt-14 'T'*** | ***conserved*** | ***conserved*** | ***conserved*** | ***pseudogene*** |
|  | ***0158-type5*** | ***59.26*** | ***52.26*** | ***63.21*** | ***nt-16 'T'*** | ***nt-9 'T'*** | ***conserved*** | ***not formed*** | ***conserved*** | ***pseudogene*** |
|  | ***0158-type6*** | ***56.94*** | ***52.26*** | ***64.02*** | ***nt-12 'A'*** | ***conserved*** | ***conserved*** | ***not formed*** | ***conserved*** | ***pseudogene*** |

**Table 3. continued....**

| **Accession name** | **Label of ITS type^a^** | **ITS1^b^** | **5.8S^b^** | **ITS2^b^** | **Motif M1^c^** | **Motif M2^c^** | **Motif M3^c^** | **Secondary structure**  **of ITS2** | **Secondary structure**  **of 5.8S** | **Note** |
| --- | --- | --- | --- | --- | --- | --- | --- | --- | --- | --- |
| **East African Highland Bananas (AAA)** |  |  |  |  |  |  |  |  |  |  |
| Ingagara (Nakitembe) | 0166-type1 | 62.50 | 57.42 | 70.05 | conserved | conserved | conserved | conserved | conserved |  |
|  | 0166-type2 | 62.04 | 57.42 | 70.05 | conserved | conserved | conserved | conserved | conserved |  |
|  | 0166-type3 | 61.57 | 57.42 | 69.91 | conserved | conserved | conserved | conserved | conserved |  |
| Mbwazirume (Nakitembe) | 1356-type1 | 62.21 | 57.42 | 70.05 | conserved | conserved | conserved | conserved | conserved |  |
|  | 1356-type2 | 61.57 | 57.42 | 70.18 | conserved | conserved | conserved | conserved | conserved |  |
|  | 1356-type3 | 62.91 | 57.42 | 70.78 | conserved | conserved | conserved | conserved | conserved |  |
| Inyoya (Musakala) | 0163-type1 | 63.89 | 56.77 | 68.84 | conserved | conserved | conserved | conserved | conserved |  |
|  | 0163-type2 | 61.57 | 57.42 | 69.91 | conserved | conserved | conserved | conserved | conserved |  |
|  | ***0163-type3*** | ***58.33*** | ***54.19*** | ***62.26*** | ***conserved*** | ***nt-9 'T'*** | ***conserved*** | ***not formed*** | ***conserved*** | ***pseudogene*** |
|  | ***0163-type4*** | ***54.63*** | ***52.90*** | ***66.36*** | ***conserved*** | ***nt-7 'T*** | ***nt-4 'A'*** | ***not formed*** | ***conserved*** | ***pseudogene*** |
|  | ***0163-type5*** | ***57.87*** | ***54.19*** | ***63.55*** | ***nt-11 'T'***  ***nt-16 'T'*** | ***nt-14 'T'*** | ***conserved*** | ***not formed*** | ***conserved*** | ***pseudogene*** |
|  | ***0163-type6*** | ***56.94*** | ***52.26*** | ***64.02*** | ***nt-12 'A'*** | ***conserved*** | ***conserved*** | ***not formed*** | ***conserved*** | ***pseudogene*** |
|  | ***0163-type7*** | ***62.04*** | ***57.42*** | ***70.18*** | ***conserved*** | ***conserved*** | ***conserved*** | ***not formed*** | ***conserved*** | ***pseudogene*** |

**^a^**) Name of ITS type contains ITC code of *Musa* accession

**^b^**) GC content [%]

**^c^**) Position of nucleotide changes (nt-) in conserved 5.8S motives
