# Supplementary figures and images for: Molecular and Cytogenetic Study of East African Highland Banana
Source: Front Plant Sci. 2018 Oct 4;9:1371. doi: 10.3389/fpls.2018.01371 (PMC6180188; doi:10.3389/fpls.2018.01371)

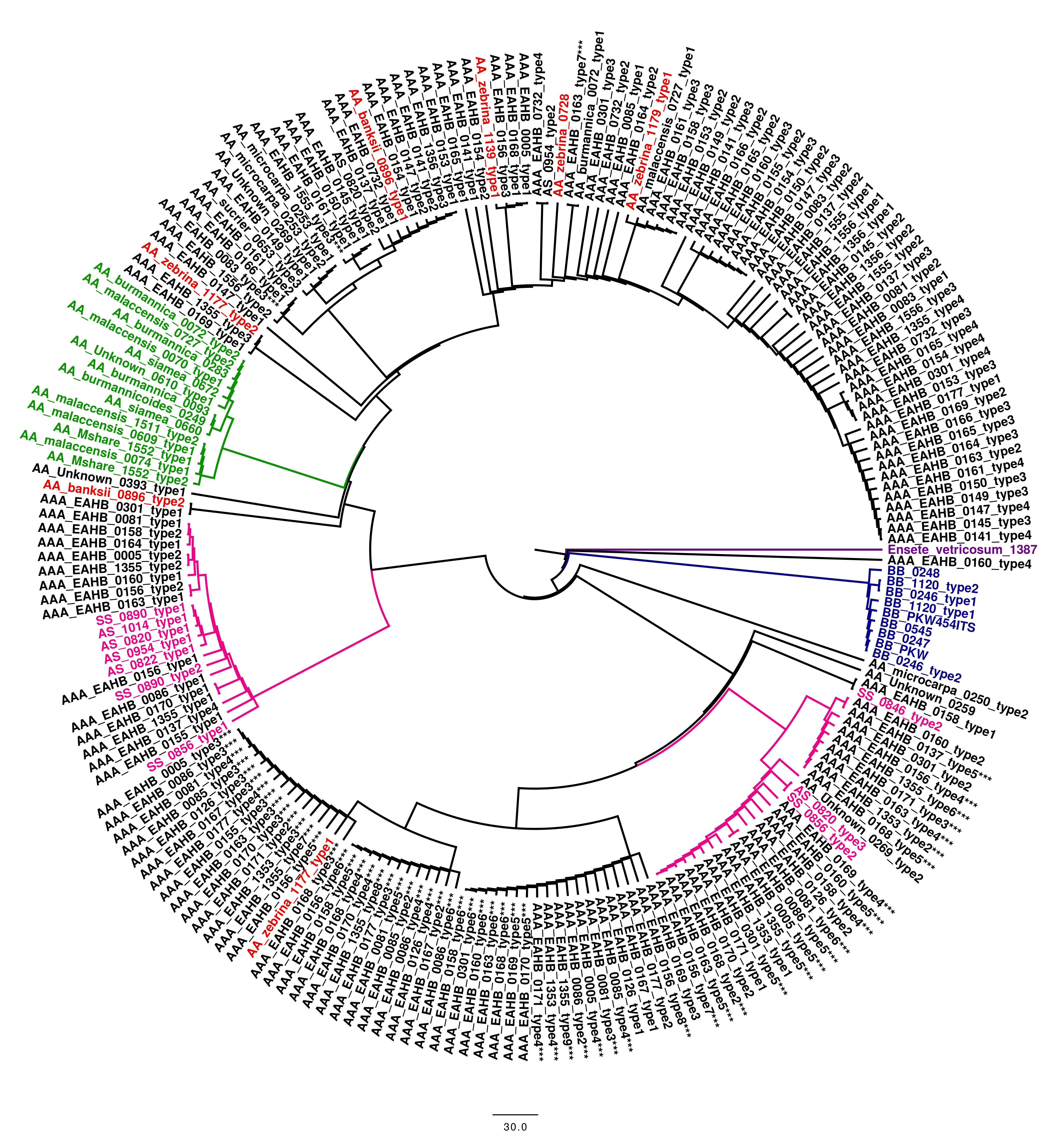

Supplement: Supplementary Figure 1 — Phylogenetic analysis based on the ITS1-ITS2 sequence region. BioNJ tree constructed from a Jukes–Cantor distance matrix of the concatenated region contained ITS1 and ITS2 spacer sequence. The tree was rooted on Ensete ventricosum (ITC 1387). The main clades and subclades are distinguished by colors: BB genotypes in blue; SS genotypes in pink; burmannica/burmanicoides/siamea and malaccensis subspecies of M. acuminata (AA genome) in green; zebrina and banksii subspecies of M. acuminata in red. Putative pseudogenic ITS sequence regions are marked by asterisk. [file Image_1.TIFF]

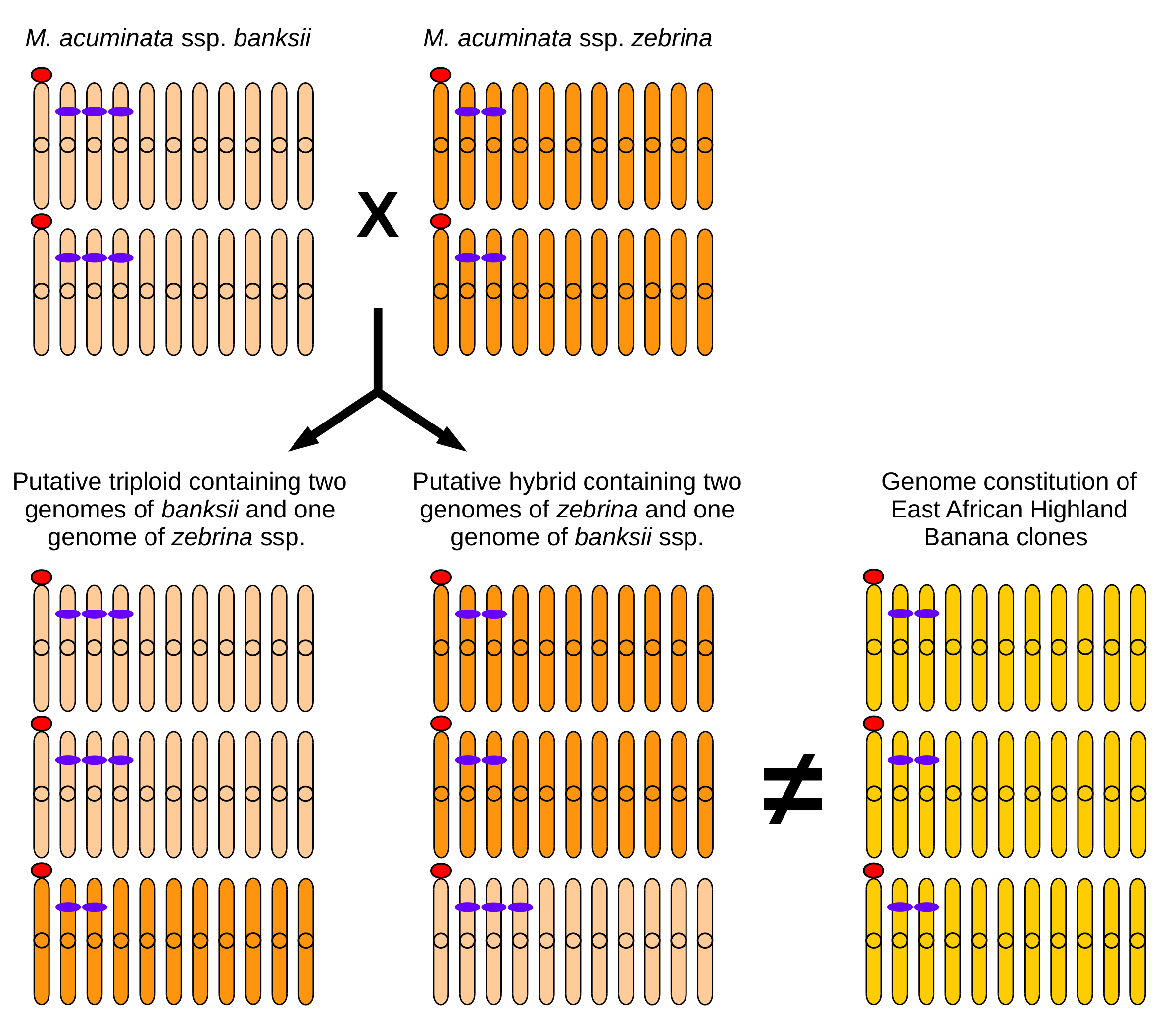

Supplement: Supplementary Figure 2 — Scheme of genomic constitution of expected banana hybrid between M. acuminata ssp. zebrina and ssp. banksii. Red and violet marks in idiograms correspond to 45S rDNA and 5SrDNA loci, respectively. [file Image_2.TIFF]
